# Acute Toxicity and Ecological Risk Assessment of Benzophenone-3 (BP-3) and Benzophenone-4 (BP-4) in Ultraviolet (UV)-Filters

**DOI:** 10.3390/ijerph14111414

**Published:** 2017-11-19

**Authors:** Yang Du, Wen-Qian Wang, Zhou-Tao Pei, Fahmi Ahmad, Rou-Rou Xu, Yi-Min Zhang, Li-Wei Sun

**Affiliations:** 1 School of Energy & Environment, Southeast University, Nanjing 210042, China; 220150552@seu.edu.cn (Y.D.); 220160595@seu.edu.cn (W.-Q.W.); 220170623@seu.edu.cn (Z.-T.P.); ahmad.fahmi@rocketmail.com (F.A.); zt746603025@gmail.com (R.-R.X.); yuran@seu.edu.cn (R.Y.); 2 Taihu Lake Water Environment Engineering Research Center (Wuxi), Southeast University, Wuxi 210042, China; 3 Research Center of Watershed Ecological Conservation and Water Pollution Control, Nanjing Institute of Environmental Sciences, Ministry of Environmental Protection of the People’s Republic of China, Nanjing 210042, China

**Keywords:** benzophenone-3, benzophenone-4, UV filters, acute toxicity, ecological risk assessment

## Abstract

Ultraviolet (UV)-absorbing chemicals (UV filters) are used in personal care products for the protection of human skin and hair from damage by UV radiation. Although these substances are released into the environment in the production and consumption processes, little is known about their ecotoxicology effects. The acute toxicity and potential ecological risk of UV filters benzophenone-3 (BP-3) and benzophenone-4 (BP-4) on *Chlorella vulgaris*, *Daphnia magna*, and *Brachydanio rerio* were analyzed in the present study. The EC_50_ values (96 h) of BP-3 and BP-4 on *C. vulgaris* were 2.98 and 201.00 mg/L, respectively. The 48 h-LC_50_ of BP-3 and BP-4 on *D. magna* were 1.09 and 47.47 mg/L, respectively. The 96 h-LC_50_ of BP-3 and BP-4 on *B. rerio* were 3.89 and 633.00 mg/L, respectively. The toxicity of a mixture of BP-3 and BP-4 on *C. vulgaris*, *D. magna*, and *B. rerio* all showed antagonistic effects. The induced predicted no-effect concentrations of BP-3 and BP-4 by the assessment factor method were 1.80 × 10^−3^ and 0.47 mg/L, respectively, by assessment factor (AF) method, which were both lower than the concentrations detected in the environment at present, verifying that BP-3 and BP-4 remain low-risk chemicals to the aquatic ecosystem.

## 1. Introduction

The dangers of excessive ultraviolet (UV) radiation to the human body has prompted the use of cosmetic products, such as sunscreen creams, lotions, etc., containing UV-absorbing chemicals (UV filters) that block UV light mainly through physical function. The most commonly used UV filters are composed of benzophenone-2 (BP-2), benzophenone-3 (BP-3), benzophenone-4 (BP-4), and 4-methylbenzylidene camphor (4-MBC), which block UV light mainly through physical function. In the production and consumption processes, these substances are released into the environment in two ways: wastewater discharge in the production process or by showering and swimming. In a study conducted in Spain, at least one kind of UV filter was detected in 51 water samples from 17 pools and hot springs [1]. In the southeastern part of Brazil, UV filters have been detected in samples from six drinking water treatment plants at different time points from June to December, where the BP-3 concentration ranged from 18.00 to 115.00 ng/L [2]. In addition, studies have shown that indoor dust also contained UV filters, where the concentrations of BP-1, BP-2, and BP-3 ranged from 80.00 to 600.00 ng/g [3]. Since the hydrophobicity and degradation rate of UV filters are poor, these compounds are likely to accumulate in the environment and produce harmful effects. Reportedly, the half maximal effective concentration at 48.h (48 h-EC_50_) of 4-MBC in the small planktonic crustacean *Daphnia magna* was 0.56 mg/L and concentrations as low as 50.00 μg/L produced negative effects on the reproduction of *D. magna* [4]. Studies have shown that UV filters impact biological reproduction. For example, BP-2 was shown to interfere with expression of the precursor egg yolk protein vitellogenin in male fathead minnows (*Pimephales promelas*) [5].

However, few studies have investigated the ecotoxicological effects of these substances, thus they have not been listed in any environmental criteria and discharge standards. Furthermore, it is reported that most processes in sewage treatment plants cannot remove UV filter chemicals effectively [6], therefore, the discharge of these chemicals is still uncontrolled. Although they are low concentration chemicals, the great mount discharging and unknown fates in the aquatic ecosystem should alarm us to pay attention to them. In our previous study [7], the acute toxic effects of Benzophenone (BP) on three species of organisms were tested and also the mixing toxic with *N,N*-diethyl-3-methylbenzamide (DEET). The acute toxicity of BP to the 3 tested organisms were classified as high or medium-level toxicity [7]. The results sparked our interests to study the toxicity of all BPs series chemicals. BPs series chemicals include 14 BP-types which are used in UV protection products [8]. Furthermore, Benzophenone (BP-1), BP-3, BP-4, 2,4,4’-Trihydroxy benzophenone (BP-8) have been frequently detected within multiple environmental matrices [9,10,11]. In the present study, the results about the toxic effects of BP-3 and BP-4 to aquatic ecosystems and the consequent environmental risk assessment are discussed.

BP-3, a broad-spectrum UV absorber, can effectively absorb UV light at 290–440 nm, but almost does not absorb visible light. BP-3 can be absorbed through the mouth and skin of humans because of its lipophilicity, light stability, and bioaccumulation [9]. As one of the most widely used UV filters, BP-3 has been detected in surface water samples collected in Hong Kong and New York, with a maximum concentration of 5429.00 ng/L in Hong Kong [10]. A recent study of endocrine disrupting effects found that BP-3 can disrupt the agonistic behavior of male Siamese fighting fish (*Betta splendens*) [12].

BP-4, a broad-spectrum UV filter with good light and thermal stability, is the most frequently detected UV filter in waste water, groundwater, and surface water in Spain. The median concentration in wastewater is 2.10 μg/L with higher concentrations in surface water and groundwater, and a maximum concentration in drinking water of 62.00 ng/L [13]. At present, there are very few studies about the toxicity of BP-4. There was only one study on the toxicity of BP-4, which found that the 48 h-EC_50_ value of *D. magna* was 50 mg/L.

The aim of this study was to investigate the ecological toxicity of BP-3 and BP-4 to aquatic organisms. The experiment employed three different freshwater species: green algae (*Chlorella vulgaris*), *D. magna*, and zebrafish (*Brachydanio rerio*). The acute ecological toxicity of the two chemicals, both singularly and in combination, were evaluated.

Based on the results of acute toxicity testing of BP-3 and BP-4, the toxic grades of these substances were determined according to the corresponding acute toxicity standards. The degree of combined toxicity was analyzed based on mixing tests. The predicted no-effect concentration (PNEC) was reduced to evaluate potential ecological risks. The results will provide a scientific basis for formulating environmental criteria and further protection of aquatic ecosystems.

All experiments were conducted in accordance with Chinese National Standards: GB/T 21805-2008 (algae) [14], GB/T 16125-2012 (*D. magna Straus*) [15], and GB/T 27861-2011 (fish) [16], as well as the Analytical Methods for Water and Wastewater [17].

## 2. Materials and Methods

### 2.1. Preparation of Chemicals

BP-3 and BP-4 at 99% purity were purchased from Aladdin Industrial Corporation (Shanghai, China). Analytical purity grade dimethyl sulfoxide (Sinopharm Chemical Reagent Co., Ltd., Shanghai, China) was used as a cosolvent.

### 2.2. Experimental Biota for Toxicity Testing

*C. vulgaris* (FACHB-8) was obtained from the Freshwater Algae Culture Collection of the Institute of Hydrobiology, Chinese Academy of Sciences (Wuhan, China). The algae were cultured for three generations in the laboratory before the toxicity experiments. Algal cells in the logarithmic growth phase were used for toxicity experiments, which were conducted in an illumination incubator to maintain the same condition: 2000–3000 lx, 25 ± 2 °C, pH 7.1, and a 12-h light: dark cycle.

*D. magna* were obtained from the Institute of Hydrobiology, Chinese Academy of Sciences, cultured in the laboratory, and then tested for sensitivity to potassium dichromate before the toxicity experiments. *D. magna* at 6–24 h old were tested. The experiments were conducted using an illumination incubator to maintain the same conditions: 25 ± 2 °C, pH 7.0–8.0, and a 12-h light: dark cycle.

*B. rerio* (AB strains; average body length, 2.60 ± 0.20 cm; average body weight, 0.33 ± 0.06 g) were obtained from Nanjing YSY Biotech Company, Ltd. (Nanjing, China). Potassium dichromate was used to test the sensitivity of *B. rerio* before the toxicity experiments. The experiments were carried out at 25 ± 2 °C, pH 7.0–8.0, and a 12-h light: dark cycle.

### 2.3. Experimental Design

#### 2.3.1. Acute Toxicity Experiments (96 h) with *Chlorella vulgaris*

Stock solutions of BP-3 and BP-4 were prepared using sterilized BG11 liquid medium. According to the results of a pre-experiment, a series of concentrations of the experimental solution (100 mL) were prepared in 250-mL glass flasks. The initial concentration of the algae in solution was 10^6^ cell/mL and the test lasted for 96 h. A blank control group and solvent control group at the highest and different concentrations were included for analysis. Three parallel samples of each group were tested. The correlation between the algal density and absorbance at 680 nm were plotted before the experiment. During the test period, the absorbance was measured every 24 h and the growth rate of algae, according to an algal density-absorbance curve, was calculated.

#### 2.3.2. Acute Toxicity Experiments (48 h) with *Daphnia magna*

Test stock solutions of BP-3 and BP-4 were prepared using aerated tap water. According to the results of the pre-experiment, a series of concentrations of the experimental solution (40 mL) were prepared in 100-mL glass beakers. Ten *D. magna* were added to each glass beaker. The test was continued for 48 h. A blank control and solvent control group at the highest concentration and different concentrations were included for analysis. Three parallel samples of each group were tested. The numbers of dead *D. magna* at every 24 h were recorded.

#### 2.3.3. Acute Toxicity Experiments (96 h) with *Brachydanio rerio*

Stock solutions of BP-3 and BP-4 were prepared using aerated tap water. According to the results of the pre-experiment, a series of concentrations of the experimental solution (4000 mL) were prepared in 5000-mL glass beakers. Ten *B. rerio* were added to each glass beaker. The test was continued for 96 h. A blank control group and solvent control group at the highest and different concentrations were included for analysis. Three parallel samples of each group were tested. The numbers of dead *B. rerio* were recorded every 24 h.

#### 2.3.4. The Mixing Toxicity Experiment

Testing of the toxicity of a mixture of BP-3 and BP-4 was conducted with the same method as for individual compounds. The mixing ratio and concentration of BP-3 and BP-4 for the different tested organisms were determined by referring to the obtained EC_50_ or 50% lethal concentration (LC_50_) of the independent acute toxicity results. 

Stock solutions of BP-3 and BP-4 were prepared for *C. vulgaris* (14 and 400 mg/L, respectively), *D. magna* (10 and 300 mg/L, respectively), and *B. rerio* (1000 and 20 mg/L, respectively). The toxic effects of the mixtures were determined by calculating the toxicity units (TU) [5] with the following formula:

(1) $$TU_{i}=\frac{C_{i}}{{\mathrm{EC}}_{50i}},$$

where C_i_ is the concentration of i in the tested solution at the EC_50_ of the mixture.

The total TU value was the sum of the *TU_i_* values of BP-3 and BP-4. If the total *TU_i_* was greater than 1, the toxicity was considered to be antagonistic, if *TU_i_* was equal to 1, the toxicity was considered to be a simple additive effect, and if *TU_i_* was less than 1, the toxicity was considered to be synergistic.

### 2.4. Classification Criteria for Acute Toxicity Test

The classification criteria for acute toxicity of BP-3 and BP-4 to the 3 species of organisms was referred to the 1) acute toxicity test classification criteria for algae (Table 1 and Table 2) acute toxicity test classification criteria for Daphnia (Table 2 and Table 3) acute toxicity test classification criteria for fish. All of the classification criteria were recorded in Analytical Methods for Water and Wastewater [17].

### 2.5. Statistical Analysis

OriginPro 9 software (OriginLab, Northampton, MA, USA) was used to draw graphs depicting the experimental results. IBM SPSS version 20 software (IBM Corp., Armonk, NY, USA) was applied to calculate the 96-h EC_50_ value of *C. vulgaris*, the 48-h LC_50_ value of *D. magna*, and the 96-h LC_50_ value of *B. rerio*.

### 2.6. Ecological Risk Assessment

AIST-MeRAM (National institute of Advanced Industrial Science and Technology-Multi-Purpose Ecological Risk Assessment and Management Tool, Japan [18]) was used to assess the ecological risks of BP-3 and BP-4 to aquatic ecosystems. PNEC values are calculated by the present toxicity data and those pre-registered in the database of AIST-MeRAM. The assessment factor (AF) and species sensitivity distribution (SSD) methods were employed to determine the PNEC. OECD recommended UF value 100 was used in the AF method. HC5 (concentration at which 5% of the species are potentially affected) was selected as an endpoint in the SSD method.

Experiments using dimethyl sulfoxide as a cosolvent and the solvent control were set at the highest concentrations in the experimental group. The results showed that there was no significant difference between the growth rate of the blank and solvent control groups, indicating that dimethyl sulfoxide has no significant effect on *C. vulgaris*, *D. magna*, and *B. rerio*, and did not interfere with the experimental results.

## 3. Results

### 3.1. Acute Toxicity of BP-3 to Aquatic Biota

#### 3.1.1. *Chlorella vulgaris* Growth Inhibition Test of BP-3

**Figure 1:**
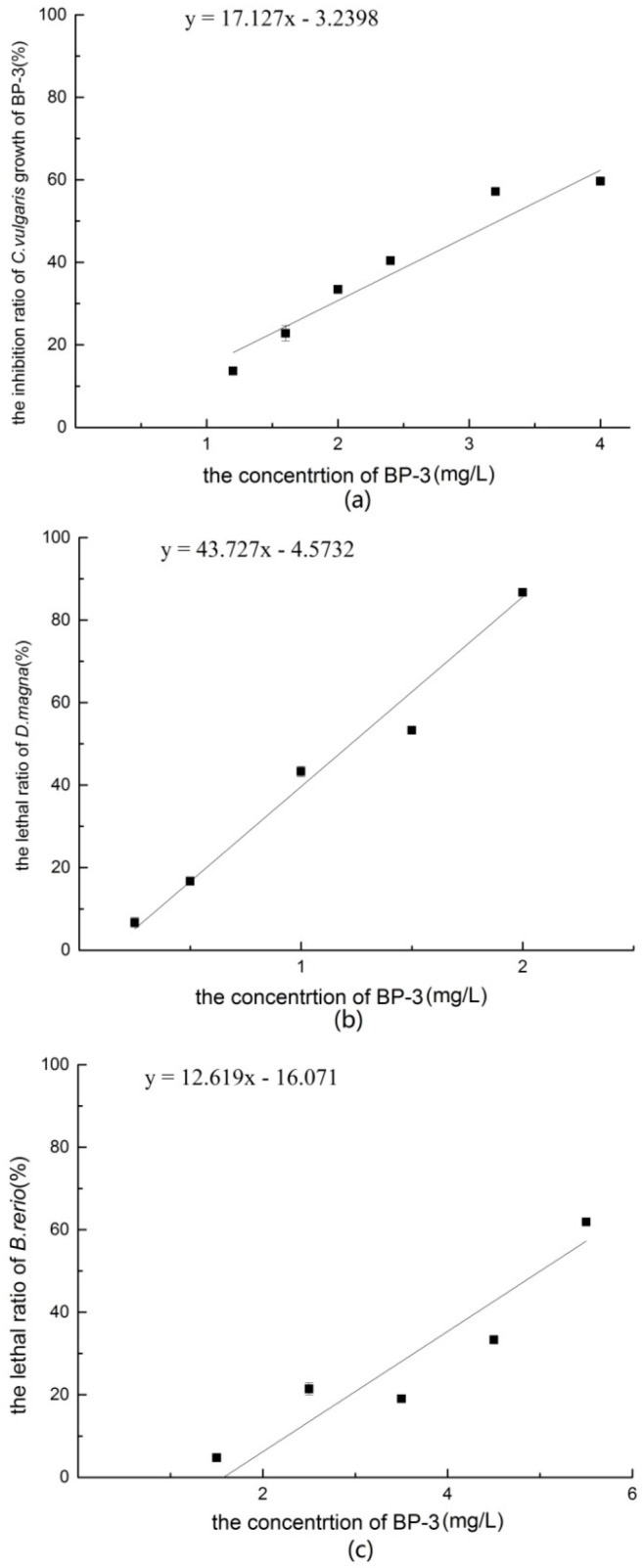
(**a**) The inhibition ratio of *C. vulgaris* growth of BP-3; (**b**) The lethal ratio of *D. magna* of BP-3; (**c**) The lethal ratio of *B. rerio* of BP-3.

When the concentration of BP-3 was lower than 2.40 mg/L, growth inhibition of *C. vulgaris* increased with the BP-3 concentration. During the exposure period, although the growth of *C. vulgaris* was inhibited, the density of algae continued to increase. At a BP-3 concentration of 4 mg/L, density of *C. vulgaris* grew rapidly for 0–48 h, but then almost stopped at 48–96 h. The relationship between the growth inhibition rate of *C. vulgaris* and the concentration of BP-3 is shown in Figure 1a. In the experimental concentration range, the effect-dose relationship was linear, as determined with the following linear regression equation:

(2) y=17.13x−3.24, (x:1.20−4.00 mg/L)

where *x* is the BP-3 concentration and *y* is the inhibition ratio on *C. vulgaris* growth (%).

Probit analysis conducted with SPSS version 20 software showed that the 96 h-EC_50_ value of BP-3 to *C. vulgaris* was 2.98 mg/L (95% confidence interval [CI] = 2.70–3.39 mg/L). According to the acute toxicity test classification criteria (Table 1) [17], the toxicity of BP-3 to *C. vulgaris* was high level.

#### 3.1.2. Acute Toxicity of BP-3 to *Daphnia magna*

The mortality of *D. magna* at BP-3 concentrations of 0.25 to 3.00 mg/L is shown in Figure 1b. In the experimental concentration range, the effect-dose relationship was linear, as determined with the following linear regression equation:

(3) y=43.72x−4.57, (x:0.25−3.00 mg/L)

where *x* is the BP-3 concentration and *y* is the mortality of *D. magna* (%).

Probit analysis conducted with SPSS version 20 software showed that the 48 h-LC_50_ of BP-3 to *D. magna* was 1.09 mg/L (95% CI, 0.76–1.73 mg/L). According to the acute toxicity classification criteria (Table 2) [17], the toxicity of *D. magna* was high-level.

#### 3.1.3. Acute Toxicity of BP-3 to *Brachydanio rerio*

As shown in Figure 1c, at a BP-3 concentration of 1.50–5.50 mg/L, the effect-dose relationship was linear, as determined with the following linear regression equation:

(4) y=12.62x−16.07, (x:1.50−5.50 mg/L)

where *x* is the BP-3 concentration and *y* is the mortality of *B. rerio* (%).

Probit analysis conducted with SPSS version 20 software showed that the 96 h-LC_50_ of BP-3 to *B. rerio* was 3.89 mg/L (95% CI = 2.86–6.53 mg/L). According to the acute toxicity classification criteria (Table 3) [17], the toxicity of *B. rerio* was high-level.

### 3.2. Acute Toxicity of BP-4 to Aquatic Biota

#### 3.2.1. *Chlorella vulgaris* Growth Inhibition Test of BP-4

**Figure 2:**
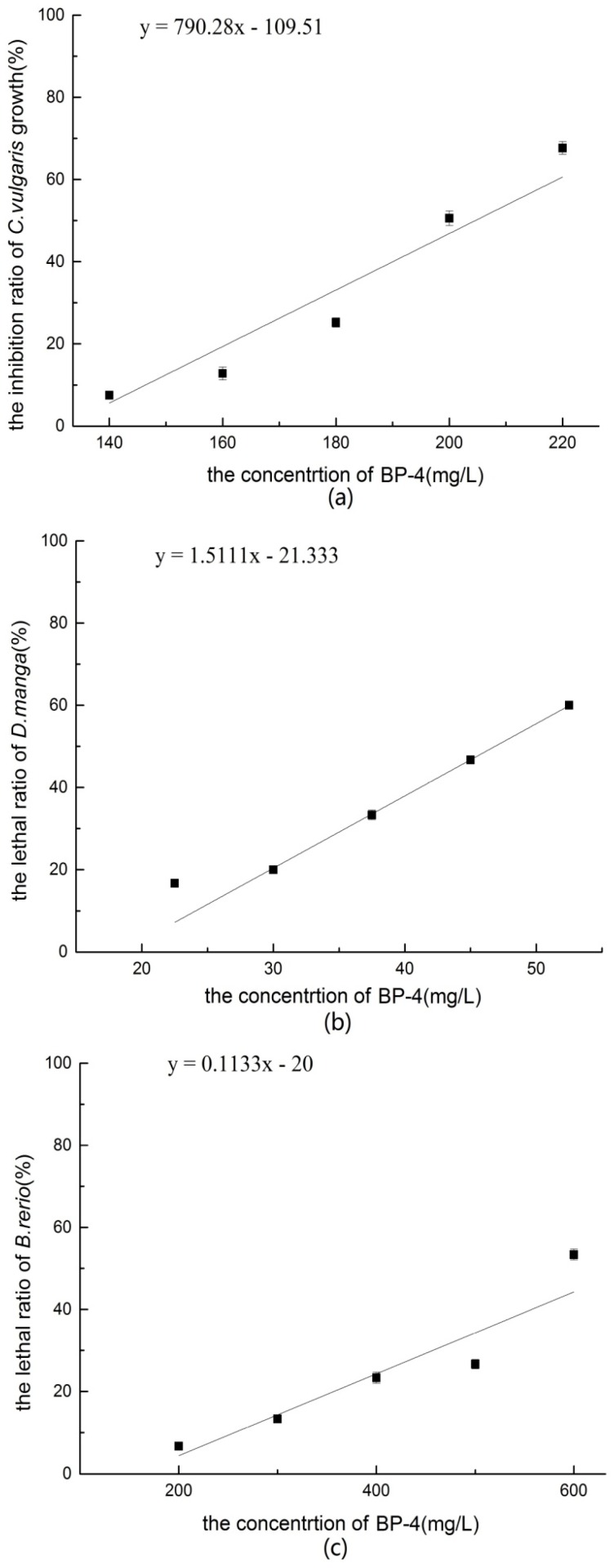
(**a**) The inhibition ratio of *C. vulgaris* growth of BP-4; (**b**) The lethal ratio of *D. magna* of BP-4; (**c**) The lethal ratio of *B. rerio* of BP-4.

As shown in Figure 2a, when the concentration of BP-4 was less than 200.00 mg/L, the inhibition *C. vulgaris* growth increased with the BP-4 concentration. During the exposure period, although the growth of *C. vulgaris* was inhibited, the density of algae had increased as a whole. When BP-4 reached 220.00 mg/L, density of *C. vulgaris* grew rapidly at 0–48 h, but then significantly slowed at 48–72 h, and almost stopped at 72–96 h. The growth rate was calculated with the following formula:

(5) y=790.28x−109.51, (x:140.00−220.00 mg/L)

where *x* is the BP-4 concentration and *y* is the inhibition ratio on *C. vulgaris* growth (%).

Probit analysis conducted with SPSS version 20 software showed that the 96 h-EC_50_ of BP-4 to *C. vulgaris* was 201.00 mg/L (95% CI = 196–209 mg/L). According to the acute toxicity classification criteria (Table 1) [17], the toxicity of *C. vulgaris* was low-level.

#### 3.2.2. Acute Toxicity of BP-4 to *Daphnia magna*

As shown in Figure 2b, at a concentration of 22.50–52.50 mg/L, the effect-dose relationship was linear, as determined with the following linear regression equation:

(6) y=1.51x−21.33, (x:22.50−52.50 mg/L)

where *x* is the BP-4 concentration and *y* is the mortality of *D. magna* (%).

Probit analysis conducted with SPSS version 20 software showed that the 48 h-LC_50_ of BP-4 to *D. magna* was 47.47 mg/L (95% CI = 36.40–654.84 mg/L). According to the acute toxicity classification criteria (Table 2) [17], the toxicity of *D. magna* was medium-level.

#### 3.2.3. Acute Toxicity of BP-4 to *Brachydanio rerio*

As shown in Figure 2c, at the highest concentration of BP-4, few *B. rerio* had survived. At a concentration of 200.00–600.00 mg/L, the effect-dose relationship was linear, as determined with the following linear regression equation:

(7) y=0.11x−20.00, (x:200.00−600.00 mg/L)

Probit analysis conducted with SPSS version 20 software showed that the 96 h-LC_50_ of BP-3 to *B. rerio* was 633.00 mg/L (95% CI = 473.00–1210.00 mg/L). According to the acute toxicity classification criteria (Table 3) [17], the toxicity of *B. rerio* was low-level.

### 3.3. Acute Toxicity of a Mixture of BP-3 and BP-4

#### 3.3.1 Acute Toxicity of a Mixture of BP-3 and BP-4 to *Chlorella vulgaris*

**Figure 3:**
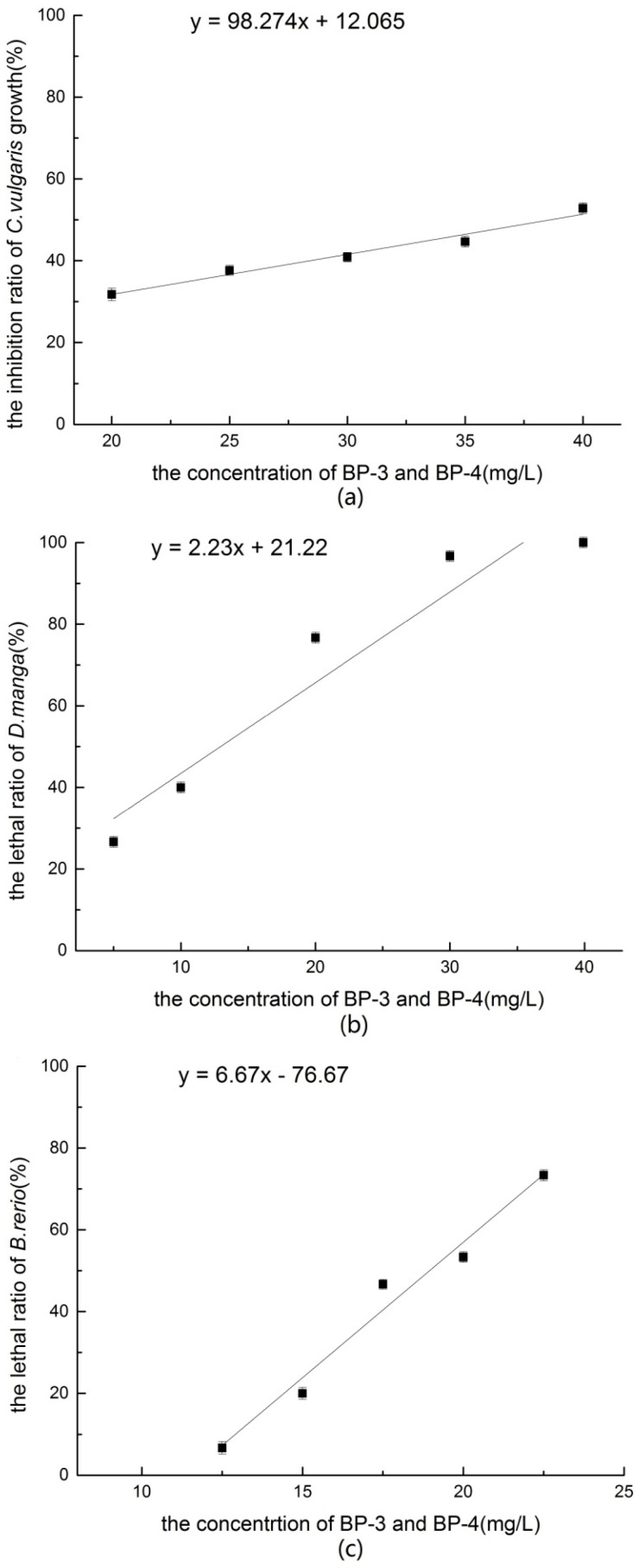
(**a**) The inhibition ratio of *C. vulgaris* growth of BP-3 and BP-4; (**b**) The lethal ratio of *D. magna* of BP-3 and BP-4; (**c**) The lethal ratio of *B. rerio* of BP-3 and BP-4.

The acute toxicity test of a mixture of BP-3 and BP-4 on *C. vulgaris* was designed according to the results of the independent acute toxicity tests. In the experiment, the growth rate of *C. vulgaris* decreased along with an increase in the concentration of the mixture. The concentration of the mixture was calculated as the percentage (%) of each chemical to the total concentration. Then, the relationship between the mixture concentration and the growth rate of *C. vulgaris* was obtained. When the percentage of the mixture was less than 30%, the density of algae had increased at 96 h. However, when the percentage of the mixture was greater than 35%, density of *C. vulgaris* almost stopped growing after 48 h. As shown in Figure 3a, the concentration of the mixture had a good linear relationship with the inhibition rate of *C. vulgaris*. The 96 h-EC_50_ value of the mixture was 44.10% (95% CI = 38.17%–57.25%). Correspondingly, the concentrations of BP-3 and BP-4 in the ∞mixture were 6.18 and 176.42 mg/L, respectively. According to the results, the calculated *TU_i_* value and the total value of the mixture were greater than 1.2. So, the toxicity of the mixture of BP-3 and BP-4 on *C. vulgaris* was antagonistic.

#### 3.3.2 Acute Toxicity of the Mixture to *Daphnia magna*

The acute toxicity of a mixture of BP-3 and BP-4 on *D. magna* is shown in Figure 3b. The mortality of *D. magna* increased along with the concentration of the mixture and showed a good dose-effect relationship. At the highest concentration of the mixture, few *D. magna* had survived, indicating that the minimum total lethal concentration was higher than the maximum concentration of the test. Probit analysis conducted with SPSS version 20 software showed that the 48 h-LC_50_ of BP-4 to *D. magna* was 9.94% (95% CI = 3.76–16.47%). Correspondingly, the concentrations of BP-3 and BP-4 in mixture were 0.99 and 29.82 mg/L, respectively. According to the results, the calculated *TU_i_* value and the total value of the mixture were greater than 1.2. So, the toxicity of the BP-3 and BP-4 mixture on *D. magna* was antagonistic.

#### 3.3.3 Acute Toxicity of the Mixture to *Brachydanio rerio*

The acute toxicity test of the mixture of BP-3 and BP-4 on *B. rerio* was designed according to the results of the independent acute toxicity tests. The acute toxicity of the mixture of BP-3 and BP-4 on B. rerio is shown in Figure 3c. In the experiment, the mortality of *B. rerio* increased along with the concentration of the mixture and showed a good dose-effect relationship. At the highest concentration used in the test, some *B. rerio* had survived, indicating that the minimum total lethal concentration was higher than the maximum concentration. Probit analysis conducted with SPSS version 20 software showed that the 96 h-LC_50_ ofBP-4 to *B. rerio* was 18.9% (95% CI = 16.8–22.7%). Correspondingly, the concentrations of BP-3 and BP-4 in the mixture were 3.78 and 189.00 mg/L, respectively. According to the calculated *TU_i_* value, the total value of the mixture was greater than 1.2. So, the toxicity of the BP-3 and BP-4 mixture on *B. rerio* was antagonistic.

### 3.4. Assessment of the Ecological Risk of BP-3 and BP-4

In this experiment, the AF and SSD methods were used to determine the PNEC. With the AF method, different countries and organizations select different UF values. In this study, the UF value according to the OECD recommendation was 100.

In addition, the SSD method used the species sensitivity curve and all possible toxicity data in the software database to analyze the toxic effects of the tested chemicals. In this study, a normal logarithm distribution model was selected and 5% was selected as the PNEC value of the species. For BP-3, the 96 h-EC_50_ value of *C. vulgaris*, the 48 h-LC_50_ value of *D. magna*, and the 96 h-LC_50_ value of *B. rerio* were input into AIST-MeRAM software. The PNEC value derived from the AF method was 0.013 mg/L (UF 100) and that derived from the SSD method was 0.11 mg/L (UF 5). For BP-4, the 96 h-EC_50_ value of *C. vulgaris*, the 48 h-LC_50_ value of *D. magna*, and the 96 h-LC_50_ value of *B. rerio* were input into AIST-MeRAM software. The PNEC value derived from the AF method was 0.47 mg/L (UF 100) and that derived from the SSD method was 29.00 mg/L (UF 5).

## 4. Discussion

### 4.1. Assessment of the PNEC Values by AF and SSD

In this study, the PNEC values of BP-3 and BP-4 were deduced by the AF and SSD methods. The PNEC values derived from the SSD method were 1–2 orders of magnitude higher than that derived by the AF method. According to the instruction to the SSD method in the software, the number and quality of toxic data have a great influence on the final results. A good accurate SSD assessment is usually defined as the results of chronic data (REACH Technical Guidance) of at least eight species. If the result was derived from SSD analysis just based on the acute toxicity data or a small amount of chronic toxicity data, the accuracy of the data is not well, and the results should be just taken as a reference value. Since studies about BPs are rare, available toxicity data remain incomplete. There were 11 and four sets of acute and chronic toxicity data, respectively, for BP-3 and BP-4 from algae, daphnia, and fish in the database of AIST-MeRAM software Therefore, the deduced PNEC values derived by the SSD method should be considered unreliable. In this case, PNEC values derived by the AF method were selected for ecological risk assessment in the present study. Because the PNEC values deduced by the AF method were lower, the environmental criteria based on this value may be overestimated. However, since presently available toxicity data are insufficient, and the SSD method cannot obtain reliable results, in order to protect the ecological environment as much as possible, the AF method was employed in the present study.

### 4.2. Acute Toxicity and Ecological Risk Assessment of BP-3

According to the individual acute toxicity results of BP-3, the 96 h-EC_50_ value of *C. vulgaris* was 2.98 mg/L, the 48 h-LC_50_ value of *D. magna* was 1.09 mg/L, and the 96 h-LC_50_ value of *B. rerio* was 3.89 mg/L. The sensitivity of *D. magna* was greater than that of *C. vulgaris* and *B. rerio*.

A query of the AIST-MeRAM database (Table 4) showed that the 72 h-EC_50_ values of BP-3 for algae were 0.67 and 0.96 mg/L, respectively (Ministry of the Environment, Japan). The sensitivity of algae to BP-3 was greater than in this study. The EC_50_ values of BP-3 to *D. magna*, as determined with the 48-h acute toxicity test, were 1.90 mg/L (Ministry of the Environment, Japan) and 1.67 mg/L [19], which were the same order of magnitude as the 48 h-LC_50_ value in this study. In addition, the 96 h-LC_50_ value of *Oryzias latipes* in the database was 3.80 mg/L, which was very close to the result of *B. rerio* in the present experiment. Based on the above data, the sensitivity of different trophic level organisms to BP-3 was Daphnia > algae > fish.

In this study, the PNEC value derived from the AF method was 1.8 × 10^−3^ mg/L. In Nanjing, BP-3 was detected in surface water at a concentration of 3.63–164 ng/L [10]. Moreover, several UV filters, including BP-3, 2-ethyl-hexyl-4-trimethoxycinnamate, and homosalate, have been detected in lakes and rivers at concentrations of 25.00, 74.00, and 92.00 ng/L, respectively. In addition, the concentration of BP-3 in the Glatt River in Switzerland was 0.1 μg/POCIS (polar organic chemical integrative samplers) from July to August 2006, and the concentration of BP-3 was up to 0.18 μg/POCIS from May to August 2017 [9]. These data indicate that the concentration of BP-3 in the aquatic environment is much lower than the deduced PNEC value. So, BP-3 may have little effect on the ecological environment at present.

However, Heffernan collected urine samples from males and females at various ages and detected BP-3 at concentrations ranging from 16.50 to 312.00 ng/L [12]. In addition, the BP-3 concentrations were greater in samples collected from adults and females, as compared to children and males. These findings may be due to the fact that adult females are more likely to use sunscreen products. Another study reported that BP-3 has an effect on endocrine function and neurodevelopment in fish [21]. Meanwhile, the concentration of BP-3 in children (aged 0–4 years) is reportedly 17.00–55.30 ng/L, which may be injurious to human health. Although very low concentrations of BP-3 have been detected in natural ecosystems, the potential for harmful effects on human health and potential endocrine effects to fish are alarming. Hence, the biotransformation process from environmental media to humans suggest potential harm of BP-3 to human health.

### 4.3. Acute Toxicity and Ecological Risk Assessments of BP-4

According to the individual acute toxicity results of BP-4, the 96 h-EC_50_ value of *C. vulgaris* was 201.00 mg/L, the 48 h-LC_50_ value of *D. magna* was 47.47 mg/L, and the 96 h-LC_50_ value of *B. rerio* was 633.00 mg/L. Based on the acute toxicity classification criteria, the toxicity of BP-4 to *C. vulgaris* and *B. rerio* were low-level, and that to *D. magna* was medium-level. The sensitivity of *D. magna* to BP-4 was greater than that of *C. vulgaris* and *B. rerio*.

At present, other than the three data sets provided by this study, there are no toxicity data for BP-4 in the AIST-MeRAM database (Table 5). There was one acute toxicity test of BP-4 to *D. magna* by Fent et al. [22], who reported a 48 h-LC_50_ value of 50.00 mg/L, which is very close to the value determined in the present study. In addition, a 14-day chronic test with the rainbow trout (*Oncorhynchus mykiss*) as the test species showed that the LOEC value was 4897.00 μg/L [11]. 

Rodil et al. [23] identified BP-4 as a potential contaminant in the water of Galicia in northwestern Spain with median concentrations of up to 2.10 μg/L in wastewater and maximum concentrations in surface water and tap water of 62.00 ng/L. In addition, the concentration of BP-4 in surface water in Tokyo, New York, and Bangkok were 71.00–136.00, 89.00–574.00, and 80.00–95.00 ng/L, respectively [20].

The PNEC value, as determined with the AF method, was 0.47 mg/L, which was significantly higher than the concentration of BP-4 detected in aquatic environments. BP-4 can be considered as an ecologically safe chemical at present.

Although the current concentration of BP-4 in the aquatic environment is low, the levels of BP-4 in the influent and outflow of a German sewage treatment plant were 2120.00 and 572.00 ng/L, respectively [24]. These concentrations did not change much and the concentration of BP-4 in sludge was 29.00 ng/g-dw. This result indicated that the sewage treatment was insufficient to effectively remove concentrated BP-4 from sludge. As a result, with the discharge of sewage, BP-4 may accumulate in the environment, thereby endangering the ecological environment and human health.

In addition, few studies have analyzed the toxicity of BP-4. We tested the acute toxicity of BP-4 on three typical organisms in the aquatic ecosystem and calculated the corresponding PNEC values. Nonetheless, future toxicity studies of different species of organisms are necessary to analyze the risks of BP-4 to the environment. 

### 4.4. Toxicity Assessment of a Mixture of BP-3 and BP-4

According to the results of the acute toxicity experiments, the effects of the mixture of BP-3 and BP-4 to *C. vulgaris*, *D. magna*, and *B. rerio* were all antagonistic. Therefore, the mixture of the two chemicals is likely to exhibit antagonism in the aquatic environment. It is worth noting that BP-3 and BP-4 both contain hydroxyl and benzophenone groups. The number and position of these hydroxyl groups are the same, with the only difference being that BP-4 contains a sulfonic acid group. Usually, an antagonistic effect is considered to be a result of similar chemical properties that play a competitive role in the active parts of the cell surface and the metabolic system, thus affecting the interactions of matter [25]. So, the similar structures of BP-3 and BP-4 exhibited a competitive role with antagonistic effects to the aquatic organisms in the present study. There has not been much discussion about the toxic mechanism of BP-3 and BP-4. It has been reported that a mixture of 4-MBC and BP-3 induced a strong increase in mRNA levels at lower concentrations with a slight induction at higher concentrations [26]. 

The toxicity of chemical mixtures is important to explain the behavior and comprehensive effects of individual chemicals in the environment. The different combinations of chemicals show different effects. In our previous studies, the mixture toxicity of BP and *N,N*-diethyl-3-methylbenzamide (DEET), on *C. vulgaris*, *D. magna*, and *B. rerio* all showed an additive effect [7], which behaved different with the combination of BP-3 and BP-4. The results of this study revealed the effects of a mixture of BP-3 and BP-4 to three trophic level organisms in an aquatic ecosystem that should help us understand the behavior and effects of these chemicals in the environment.

Based on published data, environmental concentrations and toxicity of BPs have been largely overlooked in comparison to pharmaceutical compounds. Current published literature for environmental concentrations is fairly substantial for some personal care products (PCP) compounds (ex. Triclosan, DEET, fragrances) but relatively little is available for UV filters [11]. To date, most conducted studies have investigated compounds individually but not as mixtures. The results of this study revealed the acute toxicity data of BP-3 and BP-4, and deduced the corresponding PNEC values and mixing effects. These results are significant for the assessment of the toxicity to the environmental and provide a scientific basis for critical environmental policies.

In future studies, more relevant toxicity tests, especially chronic toxicity tests, are expected to enrich the toxicity test database and assessment of chemicals used as UV filters. Continuously, all chemicals used in UV filters are expected to be evaluated. Furthermore, the potential for biomagnification and potential effects on higher trophic level organisms of these chemicals are needing to be examined, in order to protect the health of humans.

## 5. Conclusions

(1) For BP-3, the 96 h-EC_50_ value of *C. vulgaris* was 2.98 mg/L, the 48 h-LC_50_ value of *D. magna* was 1.09 mg/L, and the 96 h-LC_50_ value of *B. rerio* was 3.89 mg/L. For BP-4, the 96 h-EC_50_ value of *C. vulgaris* was 201.00 mg/L, the 48 h-LC_50_ value of *D. magna* was 47.47 mg/L, and the 96 h-LC_50_ value of *B. rerio* was 633.00 mg/L.

According to the acute toxicity classification criteria, the levels of toxicity of BP-3 on *C. vulgaris*, *D. magna*, and *B. rerio* were high. The levels of toxicity of BP-4 on *C. vulgaris*, *D. magna*, and *B. rerio* were low, medium, and low, respectively.

(2) The BP-3 and BP-4 mixture on *C. vulgaris*, *D. magna*, and *B. rerio* showed antagonistic toxic effects.
(3) By the AF method, the PNEC values of BP-3 and BP-4, were 1.80 × 10^−3^ and 0.47 mg/L, respectively, which were lower than those detected presently in the aquatic environment. These findings indicate that the ecological risk of BP-3 and BP-4 remain low.
(4) In future studies, toxicity testing of different species, especially chronic toxicity testing, should be conducted to further assess the potential ecological risks of UV filters.

## Figures and Tables

**Table 1:** Acute toxicity test classification criteria for algae.

96 h-EC_50_ (mg/L)	Toxicity Level
<1	Very high-level
1–10	High-level
10–100	Medium-level
>100	Low-level

**Table 2:** Acute toxicity test classification criteria for Daphnia.

48 h-LC_50_ (mg/L)	Toxicity Level
<1	Very high-level
1–10	High-level
10–100	Medium-level
>100	Low-level

**Table 3:** Acute toxicity test classification criteria for fish.

96 h-LC_50_ (mg/L)	Toxicity Level
<1	Very high-level
1–10	High-level
10–100	Medium-level
>100	Low-level

**Table 4:** Toxicity result of BP-3 (including the former data registered in AIST-MeRAM).

Duration Type	Endpoint	Concentration mg/L	Exposure Duration (Days)	Species	Trophic Level	Source
Acute	EC_50_^1^	1.90	2	*Daphnia magna*	Daphnids	Ministry of the Environment, Japan
Acute	LC_50_^2^	3.80	4	*Oryzias latipes*	Fish	Ministry of the Environment, Japan
Acute	EC_50_	0.67	3	Unicellular green algae (*Pseudokirchneriella subcapitata*)	Algae	Ministry of the Environment, Japan
Chronic	NOEC ^3^	0.18	3	Unicellular green algae (*Pseudokirchneriella subcapitata*)	Algae	Ministry of the Environment, Japan
Acute	LC_50_	3.90	4	*Brachydanio rerio*	Fish	This study
Acute	EC_50_	2.98	4	*chlorella vulgaris*	Algae	This study
Acute	LC_50_	1.10	2	*Daphnia magna*	Daphnids	This study
Acute	EC_50_	1.67	2	*Daphnia magna*	Daphnids	[20]
Chronic	NOEC	0.50	21	*Daphnia magna*	Daphnids	[20]
Acute	EC_50_	0.96	3	*Desmodesmus subspicatus*	Algae	[20]
Chronic	NOEC	0.13	21	*Oryzias latipes*	Fish	[20]

^1^ Concentration for 50% of maximal effect; ^2^ Lethal Concentration for 50%; ^3^ No Observed Effect Concentration.

**Table 5:** Toxicity result of BP-4 (including the former data registered in AIST-MeRAM).

Duration Type	Endpoint	Concentration mg/L	Exposure Duration (Days)	Species	Trophic Level	Source
Acute	LC_50_	633.00	4	*Brachydanio rerio*	Fish	This study
Acute	EC_50_	201.00	4	*chlorella vulgaris*	Algae	This study
Acute	LC_50_	47.46	2	*Daphnia magna*	Daphnids	This study
Acute	LC_50_	50.00	2	*Daphnia magna*	Daphnids	K. Fent et al.

## Abbreviations

The following abbreviations are used in this manuscript:

BP-3	Benzophenone-3
BP-4	Benzophenone-4
PCPs	Personal Care Products
PNEC	Predicted no effect concentration
NOEC	No Observed Effect Concentration
